# A new dataset on climate distance for trade analyses

**DOI:** 10.1016/j.dib.2024.110944

**Published:** 2024-09-17

**Authors:** Martina Bozzola, Emilia Lamonaca, Fabio G. Santeramo

**Affiliations:** a School of Biological Sciences and Institute for Global Food Security, Queen's University Belfast (UK), United Kingdom; b Research Institute of Organic Agriculture (FiBL) (CH), Switzerland; c University of Foggia (IT), Italy; d European University Institute (IT)), Italy

**Keywords:** Agri-food, Climate normal, Climate change, International trade

## Abstract

This data article describes a new dataset on measures of climate distance for trade analyses. The dataset contains, (i) time-varying measures of long-run climate conditions for twenty economies accounting for two-third of global agri-food exports and representatives of different prevailing climates, and (ii) the difference in long-run climate conditions between country-pairs, here defined as **Climate Distance**. Measures of long-run climate conditions are computed, for each year in the sample (1996 to 2015), as 30-years rolling averages of weather conditions (i.e., climate normals or climatologies) in each country. Our climate measures are based on elaboration of data collected from the World Bank's Climate Change Knowledge Portal (CCKP), containing historical information on weather conditions. We also provide data on bilateral trade flows, tariffs, and policies, as provided by publicly available datasets. Our measures of climate change allow a more precise evaluation of structural changes in trade routes associated with climate comparative advantages.

Specifications Table

**Table:** 

Subject	Economics.
Specific subject area	Climate Economics and International Trad*e*
Type of data	Raw data .txt (ASCII text files) .do file (Stata files) .dta (Stata datasets).
Data collection	Our measures of climate distance are based on existing dataset containing historical information on observed and quality-controlled temperature and rainfall values from thousands of weather stations worldwide, aggregated at the national level (source: CCKP). This data source has been selected for their high accuracy and country-time coverage. Additional data to conduct trade analyses are collected from databases on trade flows among country pairs (source: UN Comtrade), applied tariffs on imports (source: WITS), trade facilitation measures (source: CEPII).
Data source location	The sources of raw data used to estimate the trade effects of climate distance are hosted at University of Foggia: via Napoli, 25 - 71,121 Foggia, Italy
Data accessibility	Repository name: Mendeley Data Data identification number: DOI: 10.17632/b54ktpthrp.1 Direct URL to data: https://data.mendeley.com/datasets/b54ktpthrp/2
Related research article	Bozzola, M., Lamonaca, E., & Santeramo, F. G. (2023). Impacts of climate change on global agri-food trade. Ecological Indicators, 154, 110,680. https://doi.org/10.1016/j.ecolind.2023.110680

## 1 Value of the Data

• The evolution of the climate conditions alters countries’ comparative advantages.
• The differences in climate, defined as climate distances, help assessing and modelling bilateral trade.
• Climate distance complements and expands the set of variables germane geographical features.

## 2 Background

Climate change alters comparative advantages and trading opportunities. Climate distance, defined as absolute difference between climate normals, is a driver of bilateral trade. This paper adds to the related research papers [1,2] by providing a careful discussion of the measures of climate distance and the associated database.

## 3 Data Description

This data article, along with the related research articles [1,2], provide two datasets of climate normals (Ricardian Dataset) and climate distance (Gravity Dataset) for twenty economies, listed in Table 1. Our sample ensures representativeness in terms of geographical location (low-latitude and high-latitude regions) and prevailing climate.

**Table 1:** List of countries in the sample.

Country	ISO 3	Region	Hemisphere
Argentina	ARG	Latin America and Caribbean	Southern
Australia	AUS	East Asia and Pacific	Southern
Brazil	BRA	Latin America and Caribbean	Southern
Canada	CAN	North America	Northern
China	CHN	East Asia and Pacific	Northern
Germany	DEU	Europe and Central Asia	Northern
Spain	ESP	Europe and Central Asia	Northern
France	FRA	Europe and Central Asia	Northern
United Kingdom	GBR	Europe and Central Asia	Northern
Indonesia	IDN	East Asia and Pacific	Southern
India	IND	South Asia	Northern
Israel	ISR	Middle East and North Africa	Northern
Italy	ITA	Europe and Central Asia	Northern
Jordan	JOR	Middle East and North Africa	Northern
Morocco	MAR	Middle East and North Africa	Northern
New Zealand	NZL	East Asia and Pacific	Southern
Peru	PER	Latin America and Caribbean	Southern
Russia	RUS	Europe and Central Asia	Northern
United Stated	USA	North America	Northern
South Africa	ZAF	Sub-Saharan Africa	Southern

The complete datasets are made available in ASCII text and in STATA (.dta) formats and can be accessed through a dedicated Mendeley repository [3]. Beyond the datasets on climate normals (“Dataset_Ricardian_Bozzola_Lamonaca_Santeramo.txt”, “Dataset_Ricardian_Bozzola_Lamonaca_Santeramo.dta”) and climate distance (“Dataset_Gravity_Bozzola_Lamonaca_Santeramo.txt”, “Dataset_Gravity_Bozzola_Lamonaca_Santeramo.dta”), we also provide in the Mendely repository an ASCII text file (“Code_Bozzola_Lamonaca_Santeramo.txt”) and a STATA .do file (“Code_Bozzola_Lamonaca_Santeramo.do”) that will guide the user to replicate findings in Bozzola, Lamonaca and Santeramo [1] and in Lamonaca, Bozzola and Santeramo [2].

The variables contained in the datasets are listed and briefly described in Table 2. In the Ricardian Dataset, for each exporter *i*, we provide (i) annual averages and climate normals or climatologies (i.e., 30-years averages) of temperature and precipitation and their squares – variables name: t, p, temp, prec, temp_2, prec_2; (ii) total exports of ‘Food and beverages’ aggregated at the one-digit level of the classification by Broad Economic Categories (BEC 1996: 01) in level and logarithm (variable names: exports, lnexp); (iii) country-specific information, such as geographical location (variable name: region_i), latitude/longitude coordinates defining the centre point of a country (variables name: lat_i, lon_i), development status (variable name: dc_i). In the Gravity Dataset, for each pair of exporter *i* and importer *j*, we provide (i) the absolute difference between annual average climate and between climate normals of trading partners (i.e., climate distance) and their logarithm – variables name: abs_t_ij, abs_temp_ij, ln_abs_temp_ij, abs_p_ij, abs_prec_ij, ln_abs_prec_ij; (ii) bilateral exports of ‘Food and beverages’ aggregated at the one-digit level of the classification by Broad Economic Categories (BEC 1996: 01) in level (variable name: exports); (iii) bilateral determinants of trade, such as the membership in Regional Trade Agreement (variable name: rta), the presence of Sanitary and Phytosanitary (SPS) measures (variable name: sps_bil_dummy), simple average tariff (variable name: ln_tariff), the geographical distance multiplied by a time trend (variable name: ln_dist_trend), the membership in the World Trade Organization (variable name: wto).

**Table 2:** Variable description.

Dataset	Variable name	Type	Description
Ricardian	year	Number	Time identifier (years: 1996–2015)
	y30	String	30-years period used to build climate normals
	i	String (encoded)	Panel identifier, ISO3 alphanumeric code of exporter
	region_i	String	Region of exporter
	dc_i	Categorical (0/1)	Equal to one if the exporter is a developed country
	lat_i	Number	Latitude of exporter (Decimal Degrees)
	lon_i	Number	Longitude of exporter (Decimal Degrees)
	exports	Number	Total exports (Thousand USD)
	lnexp	Number	Natural logarithm of total exports (ln thousand USD)
	t	Number	Average annual temperature (°C)
	p	Number	Average annual precipitation (mm)
	temp	Number	30-years rolling average annual temperature (°C)
	prec	Number	30-years rolling average annual precipitation (mm)
	temp_2	Number	Square of 30-years rolling average annual temperature (°C^2^)
	prec_2	Number	Square of 30-years rolling average annual precipitation (mm^2^)
Gravity	year	Number	Time identifier (years: 1996–2015)
	y30	String	30-years period used to build climate normals
	ij	Numerical	Panel identifier
	i	String (encoded)	ISO3 alphanumeric code of exporter
	dc_i	Categorical (0/1)	Equal to one if the exporter is a developed country
	j	String (encoded)	ISO3 alphanumeric of importer
	exports	Number	Bilateral exports (million USD)
	abs_t_ij	Number	Absolute difference in temperature of exporter and importer (°C)
	abs_temp_ij	Number	Absolute difference in temperature normals of exporter and importer (°C)
	ln_abs_temp_ij	Number	Natural logarithm of absolute difference in temperature normals of exporter and importer (ln °C)
	abs_p_ij	Number	Absolute difference in precipitation of exporter and importer (mm)
	abs_prec_ij	Number	Absolute difference in precipitation normals of exporter and importer (mm)
	ln_abs_prec_ij	Number	Natural logarithm of absolute difference in precipitation normals of exporter and importer (ln mm)
	rta	Categorical (0/1)	Equal to one if the exporter and importer are members of a Regional Trade Agreement
	sps_bil_dummy	Categorical (0/1)	Equal to one if Sanitary and Phytosanitary (SPS) measures are in place
	ln_tariff	Number	Natural logarithm of simple average tariff
	ln_dist_trend	Number	Natural logarithm of geographical distance multiplied by a time trend
	wto	Categorical (0/1)	Equal to one if the exporter and importer are members of the World Trade Organization
	it	Numerical	Numerical code for combinations of importer-time
	jt	Numerical	Numerical code for combinations of exporter-time
	pair	Numerical	Numerical code for combinations of exporter-importer

**Fig. 1:**
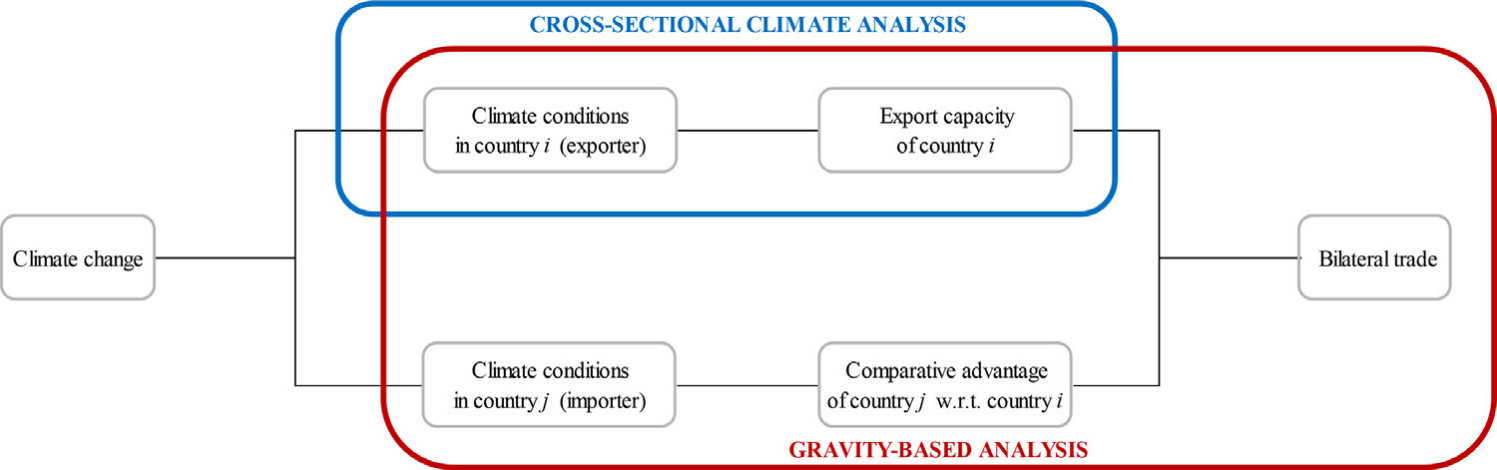
Experimental design.

*Source*: Elaboration on Figure B.1 in Bozzola, Lamonaca and Santeramo [1].

In our sample, the average temperature normal is 13.6 °C and precipitation normal is 73.4 mm. In Table 2 and Fig. 5 in Bozzola, Lamonaca, Santeramo [1], we split the evolution of 30-years average annual temperatures and precipitations for developed and developing trading partners. The climate distance is highly heterogenous across space (between country-pairs) and slightly variable across time (within country-pairs), as shown in Table 1 and Fig. 1 in Lamonaca, Bozzola, Santeramo [2]. We show the five largest absolute distances in long-term average temperature and precipitation between trading partners for the period 1986–2015 (Tables 3 and 4).

**Table 3:** Top 5 temperature distant trading partners ( °C), 1986–2015.

Notes: Acronyms are Brazil (BRA), Canada (CAN), Indonesia (IDN), India (IND), Russia (RUS).

**Table 4:** Top 5 precipitation distant trading partners (mm), 1986–2015.

Notes: Acronyms are Indonesia (IDN), Israel (ISR), Jordan (JOR), Morocco (MAR), Russia (RUS).

## 4 Experimental Design, Materials and Methods

Our experimental design postulates that the climate change is reflected in the climate normal of the trading partners and alters their comparative advantages. The evolution of comparative advantages is reflected in countries’ trade capacity (Fig. 1, blue box). The divergences in climate conditions (referred to as Climate Distance) induce changes in bilateral trade due to different specialization incentives (Fig. 1, red box) (Tables 3 and 4).

In the first part of the analysis, we build upon cross-sectional climate studies (e.g., [4,5]) and we implicitly assume that countries can adapt to changes in climate ([6]). This approach is a popular hedonic method for analyzing climate change impacts on agriculture ([7]). We use this approach to estimate how much climate explains observed cross-sectional variation of the value of countries’ agri-food trade (land value in the original application of this approach), controlling for confounding factors. This cross-sectional method was developed to study the long-term impacts of climate change on agriculture while accounting for adaptation ([4]). The strength of the method is its ability to measure the long run impacts of climate change considering the ability of each farmer to adapt. However, the method does not analyze how adaptation is implemented, and it does not consider future changes in crop varieties and animal breeds, technology, prices, and investments. It is not known how these other changes might affect the climate sensitivity of farms.

We implicitly assume that each trading partner has adapted to the climate it currently faces, and the result reflects this adaptation. The result of a general cross-sectional model describes the net outcome across the entire agricultural system. Each agricultural area, having adopted a specific crop and type of farming is captured along this function. If conditions change substantially, the function describes what would happen to that land if it changed into a different production system.

We translate the baseline concepts of cross-sectional climate studies into a gravity-type setting (e.g., [8,9]) to link the evolving trade patterns to climate change adaptation strategies. We define ‘climate distance’ as the difference between climate of trading partners. Insofar climate distance alters comparative advantages, it affects production, process and trade decisions, as reflected by international shipments. In other terms, climate distance influence trade and the evolution of value chains.^1^ Changes in climate distance is, therefore, a driver of bilateral trade. This novel measure of distance between trading partners, based on the difference in their climate conditions, complements and expand the set of bilateral distance traditionally used in gravity models: i.e., geographical, cultural, geopolitical distance.

We focus on the long-term impacts of climate change, and not on the short-term impacts of weather shocks. The effect of weather is conceptually different from the effect of climate due to the adaptation efforts of farmers, regions, and countries ([12,13]). In climate analyses it is common to use climate normals or climatologies (i.e., 30-years averages) of weather conditions. We compile historical data from the widely adopted Climate Change Knowledge Portal (CCKP) of the World Bank ([14,15]). We referred to the Climatic Research Unit (CRU) Time Series (TS) Version 3.10 Dataset ([16]). The gridded historical dataset is derived from observational data and provides quality-controlled temperature and rainfall values from thousands of weather stations worldwide, presented at a spatial resolution of 0.5° latitude by 0.5° longitude grid (approximately 55 km by 55 km at the equator) over all land domains^2^ and aggregated at the national level by the CCKP. We collect annual country-means for temperature and the cumulative value of yearly precipitation,^3^ established over the respective time windows, from 1961 to 2015. This long period allows us to build climate normals of temperature and precipitation, based on 30-years rolling averages, for each year in our sample (from 1996 to 2015). For instance, the climate normal of temperature in 1996 is obtained as the average of annual mean values for temperature in 1967–1996. The climate normal of temperature of the following year is the average over the period 1968–1997. The value in the last year of the sample is the average of annual mean values for temperature in 1986–2015.

Trade data are from the United Nations Comtrade Database, the world's most comprehensive global trade data platform [18]. Data on the membership in Regional Trade Agreements are collected from the Gravity Database of the Centre d'Etudes Prospectives et d'Informations Internationales (CEPII) [19]. Information on the presence of SPS measures are retrieved from Santeramo and Lamonaca [20]. Simple average tariffs are downloaded from the World Bank's World Integrated Trade Solution (WITS) Database [21].

## Limitations

Not applicable.

## Ethics Statement

The current work does not involve human subjects, animal experiments, or any data collected from social media platforms.

## CRediT Author Statement

Authors are listed in alphabetical order. **Martina Bozzola**: Conceptualization, Data curation, Methodology, Validation, Writing- Reviewing and Editing. **Emilia Lamonaca**: Conceptualization, Investigation, Data curation, Methodology, Validation, Software, Visualization, Writing, Original draft preparation. **Fabio G. Santeramo**: Conceptualization, Investigation, Methodology, Validation, Supervision, Writing- Reviewing and Editing.

## Data Availability

Dataset on climate distance for trade analyses (Original data) (Mendeley Data).

## Footnotes

1 Understanding how climate distance impacts, through trade costs, the evolution of domestic and international agricultural value chains is a promising area of research [10,11].

2 There is no distinction between agricultural and non-agricultural land areas.

3 Climate conditions may be captured by several indicators, other than the long-term average of temperature and precipitation. Some examples include sunshine duration, relative humidity, and wind speed. Other indicators are not included in this dataset due to limited data availability: data on sunshine duration are available for 65 % of the sample, data on relative humidity and wind speed are available for 35 % of the sample (but countries in these subsamples are different); less than 20 % of the sample shares data on sunshine duration, relative humidity, and wind speed [2]. Other indicators of climate conditions are crop specific and difficult to quantify for each traded commodity. Examples of these are critical values of temperature and precipitation depending on the growing phases, drought index, occurrence of extreme events [17].
